# Dark Sweet Cherry (*Prunus avium*) Supplementation Reduced Blood Pressure and Pro-Inflammatory Interferon Gamma (IFNγ) in Obese Adults without Affecting Lipid Profile, Glucose Levels and Liver Enzymes

**DOI:** 10.3390/nu15030681

**Published:** 2023-01-29

**Authors:** Shirley Arbizu, Susanne U. Mertens-Talcott, Stephen Talcott, Giuliana D. Noratto

**Affiliations:** Department of Food Science and Technology, Texas A&M University, College Station, TX 77843, USA

**Keywords:** dark sweet cherries, obesity, inflammation, metabolic disorders, clinical study

## Abstract

Dark sweet cherries (DSC) are rich in fiber and polyphenols that decrease risk factors associated with obesity. This single-blind randomized placebo-controlled study investigated DSC effects on inflammation, cardiometabolic, and liver health biomarkers in obese adults. Participants (>18 years, body mass index (BMI) = 30–40 kg/m^2^) consumed 200 mL of DSC drink (juice supplemented with DSC powder) (*n* = 19) or a placebo drink (*n* = 21) twice/day for 30 days. Anthropometric and physiological biomarkers were monitored at baseline (D1), mid-point (D15), and endpoint (D30) visits. Blood inflammatory biomarkers were assessed at D1, D15, and D30, and blood lipids, glucose, and liver enzymes at D1 and D30. DSC consumption lowered systolic blood pressure (SBP) (*p* = 0.05) and decreased diastolic blood pressure (DBP) compared to placebo (*p* = 0.04). Stratification of participants by BMI revealed a greater (*p* = 0.008) SBP reduction in BMI > 35 participants. DSC lowered pro-inflammatory interferon-gamma (IFNγ) (*p* = 0.001), which correlated with SBP changes. The interleukin (IL)-1RA and SBP changes were correlated in the placebo group, as well as triglycerides (TG) with DBP. The increased IL-10 levels in the placebo group suggested a compensatory mechanism to counteract elevated IFNγ levels. No significant between-group differences were detected for blood lipids, glucose, and liver enzymes. In conclusion, DSC helped to decrease blood pressure levels and inflammation in obese adults.

## 1. Introduction

Obesity is a global epidemic that represents a major risk factor for cardiovascular diseases (CVD), type 2 diabetes (T2D) and some types of cancer [1]. According to the Center for Disease Control and Prevention (CDC), the prevalence of obesity has grown exponentially over the last decade in the U.S. and now affects almost 31% of the total adult population [2]. Obesity is associated with an increased risk for developing metabolic syndrome, a cluster of conditions characterized by abdominal obesity, hyperglycemia, dyslipidemia, hypertension and altered cholesterol levels [3]. These abnormalities appear to be caused by oxidative stress and systemic inflammation [3]. 

Oxidative stress, mediated by the overproduction of reactive oxygen species (ROS), induces cellular damage and promotes the activation of inflammatory signaling cascades [4]. This scenario is reflected in elevated systemic concentrations of pro-inflammatory molecules which are also implicated in the development of obesity-related complications, including CVD and T2D [5,6]. More specifically, elevated concentrations of pro-inflammatory cytokines (e.g., interleukin (IL)-6, -18, -1β, tumor necrosis factor alpha (TNF-α), monocyte chemoattractant protein 1 (MCP-1)), as well as increased levels of C-reactive protein (CRP) have been reported in overweight and obese adults [5,6]. Moreover, obesity often results in liver diseases such as non-alcoholic fatty liver disease (NAFLD) which is characterized by the accumulation of fat in the liver (hepatic steatosis), resulting in liver inflammation, fibrosis, cirrhosis and hepatocellular carcinoma [7,8]. 

Given the significant role of nutrition in the progression and sustenance of obesity, numerous studies support the inverse association between a diet rich in fiber and polyphenols, and obesity along with its associated metabolic disorders [9]. Dark sweet cherries (*Prunus avium*) (DSC) are appreciated by consumers due to their organoleptic attributes and content of bioactive compounds including polyphenols [10]. DSC are considered a relevant source of phenolic acids and anthocyanins, with the latter being responsible for the deep red color characteristic of the fruit. Evidence from animal models have shown that DSC polyphenols can exert protective effects against metabolic complications associated with obesity. For example, diet supplementation with DSC anthocyanins (40 mg and 200 mg/kg × 12 weeks) resulted in lowered serum glucose, liver triglycerides, total cholesterol, and LDL-cholesterol in mice fed a high-fat diet [11]. Moreover, we have previously reported that non-anthocyanin phenolics and fiber in DSC powder decreased biomarkers of liver disease and lessened plasma pro-inflammatory IL-6 in obese-diabetic mice [12]. Human studies have demonstrated that DSC consumption (280 g/day) for 28 days lowered circulating concentrations of inflammatory markers such as CRP, IL-18 and regulated upon activation, normal T-cell expressed, and secreted (RANTES) in healthy individuals [13,14]. In addition, a pilot cross-over study found that anthocyanin-rich DSC juice (300 mL) resulted in significant reduction in blood pressure and heart rate after 2 h consumption in young and older adults [15]. Findings from these studies suggest that DSC supplementation could exert anti-inflammatory effects relevant to the prevention of obesity-related comorbidities. However, to our knowledge, no previous studies have evaluated the effects of DSC intake in obese individuals. The goal of this study was to investigate if DSC supplementation modulates levels of inflammatory and metabolic biomarkers in obese individuals using a placebo-controlled single blind randomized study design. 

## 2. Materials and Methods

### 2.1. Study Design and Participant Eligibility

This study was conducted in accordance with the Declaration of Helsinki, and the protocol was approved by the Institutional Review Board (IRB2019-0597F) at Texas A&M University. This study was registered at clinicaltrials.gov as NCT05586386. Participants were recruited between January 2020 and August 2021 according to the following inclusion criteria: Age: ≥18 years old, body mass index (BMI): ≥30 and ≤40, without a history of chronic diseases or intestinal disorders, and with a willingness to stop taking nutritional supplements. Participants were excluded if they had any of the following conditions within the previous six months: acute cardiac event, stroke, cancer, alcohol or substance abuse, hepatitis (B or C) or HIV, liver or renal dysfunction, history of dizziness/fainting during and after blood draws, lactose intolerance, gluten sensitivity or celiac disease. Excessive drinking, allergy or sensitivity to berries, pregnancy/lactation and smoking were also considered exclusion criteria. Eligible subjects were contacted to participate in a familiarization session in which detailed information of the study along with the consent form were presented. The use of medication as exclusion criteria was modified halfway throughout the study to increase recruitment. Each participant provided a written consent form before any study procedure was performed. Screening visits were scheduled to collect anthropometric (weight, height, BMI, and waist circumference) and physiological measurements (blood pressure, heart rate and oxygen saturation). During screening visits, participants also completed a voluntary demographic questionnaire. After completion of a 2-week run-in period, in which participants were given instructions to refrain from intake of polyphenol-rich foods and nutritional supplements, allocation to cherry or placebo groups was based on covariate adaptive randomization. 

**Figure 1:**
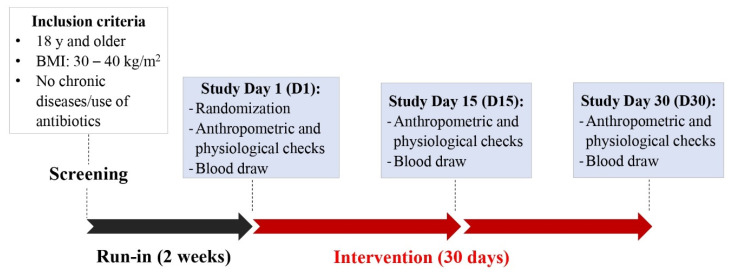
Graphical representation of study protocol.

This randomization method minimizes the imbalance between groups by considering specific covariates and previous assignments of participants [16]. The stratification was performed based on prognostic variables: BMI (low: BMI between 30 and 35, high: BMI between 35 and 40), and gender (male and female). Three study visits were scheduled after the run-in period to collect data from baseline or day 1 (D1), halfway of intervention or day 15 (D15) and at the end of the study or day 30 (D30), as shown in Figure 1. 

### 2.2. DSC Supplementation

DSC concentrated juice, kindly provided by FruitSmart^®^ (Grandview, WA, USA), was supplemented with DSC powder, kindly provided by Anderson Advanced Ingredients (Irvine, CA, USA). DSC concentrated juice and DSC powder were stored at −20 °C and room temperature, respectively. The characteristics of DSC concentrated juice, DSC powder, and reconstituted DSC drink are presented in Supplementary Table S1. The placebo concentrated juice was prepared by study personnel following good manufacturing practices in the Department of Food Science and Technology at Texas A&M University and stored at −20 °C. Placebo reconstituted drink matched °Brix, color, and organoleptic characteristics of DSC reconstituted drink. Formulation of placebo concentrated juice and nutritional and physicochemical characteristics are presented in Supplementary Table S2. 

Participants were given instructions to reconstitute DSC or placebo concentrated drinks and consume twice/day for 30 days without modifying their dietary patterns and physical activity. The reconstituted DSC drink contained 50 mL DSC concentrated juice, 3 g DSC powder and 150 mL water. In this study, DSC beverage containing DSC juice and powder was meant to be used as a supplementary fruit drink to supply phytochemicals and some fiber from the whole fruit to participants in a consistent and standardized presentation for easy compliance. The 400 mL total volume DSC drink intake per day exceeded the recommended 150 mL fruit juice per day which can count as 1 portion of the recommended five daily portions of fruit and vegetables. Drinking more than 150 mL fruit juice a day over an extended period may be linked to body weight gain due to the sugar content. Therefore, eating the whole fruit may be a healthier option because of dietary fiber and other nutrients and phytochemicals in skin and pulp. Treatment compliance was assessed with the daily self-intake report that was returned to the study coordinator at the end of the intervention. Participants were given verbal and written instructions to continue refraining from polyphenol-rich foods and nutritional supplements as in the run-in period and to refrain from berry intake during the study. 

### 2.3. Dietary Assessment

Nutritional patterns were evaluated using 15-day dietary records collected during the 30-day intervention. Participants were instructed to use Myfitnesspal (www.myfitnesspal.com (accessed by participants between January 2020 and August 2021) to record their daily food and beverage intake. Diet quality was assessed according to the 2015-Healthy Eating Index (HEI), which is an indicator of the adherence to the Dietary Guidelines for Americans (DGA) [17]. Thirteen components, based on servings per 1000 kcal, were used to calculate HEI scores with values of 0 and 100 indicating the lowest and highest adherence to the DGA, respectively [17]. 

### 2.4. Anthropometric Measurements and Physiological Biomarkers

At each study visit, participants underwent anthropometric (body weight, height, BMI, waist, and hip circumference), and physiological measurements (temperature, systolic and diastolic blood pressure, heart rate and oxygen saturation), and were asked if any health symptoms were observed as result of intervention. Body weight (BW, kg) and height (m) measurements were performed using a digital scale (Health-o-meter, Boca Raton, FL, USA) and recorded to the nearest units. BMI (kg/m^2^) and body fat content (BF, %) were determined using a handheld fat loss monitor (Omron Healthcare, Bannockburn, IL, USA). Waist circumference (WC, cm) was measured to the nearest millimeter. Systolic and diastolic blood pressure (SBP and DBP, respectively, mmHg) were measured thrice after approximately 5 min of rest, using a blood pressure monitor (Omron Healthcare, Bannockburn, IL, USA). Heart rate (HR, bpm) and oxygen saturation (OS, SpO_2_%) were recorded thrice using a fingertip pulse oximeter (Santamedical, Tustin, CA, USA). 

### 2.5. Blood Sample Collection

Participants were instructed to fast for 12 h before each study appointment and to drink plenty of water to facilitate blood draws. Blood samples (approximately 10 mL) were collected in EDTA-coated tubes and centrifuged at 2000 rpm at 4 °C to separate plasma and red blood cells (RBC). Blood fractions were aliquoted and stored at −80 °C for further analysis. 

### 2.6. Analysis of Inflammatory and Oxidative Stress Biomarkers

Cytokines. Plasma samples collected on D1, D15 and D30 were used to assess the levels of cytokines using the multiplex bead-based immunoassay Luminex system using the Milliplex^®^ MAP Human Cytokine/Chemokine/Growth Factor panel (EMD Millipore, Billerica, MA, USA) according to the manufacturers’ protocol. Analytes included: Interleukin (IL)-1 receptor antagonist (RA), IL-18, IL-6, IL-1β and IL-10, interferon gamma (IFNγ), TNF-α, RANTES and MCP-1. Data was analyzed using the Luminex xPonent 3.1 software.

Erythocyte sedimentation rate (ESR): Inflammatory conditions were monitored though the ESR test using the Sedi-Rate ESR System (Globe Scientific Inc., Mahwah, NJ, USA) following the manufacturer’s instructions on blood samples (approximately 5 mL) collected in Vacutainer^®^ EDTA tubes (Franklin Lakes, NJ, USA) within 1 h of blood draws on D1 and D30. 

C-reactive protein (CRP): CRP was quantified in plasma samples collected on D1, D15 and D30 using the Human Neurodegenerative Disease Magnetic Bead Panel 2 (EMD Millipore; Billerica, MA) following the manufacturers’ instructions. Data was analyzed using the Luminex xPonent 3.1 software. 

White blood cell (WBC) count differential test: Blood samples (approximately 5 mL) collected on D1 and D30 in Vacutainer^®^ EDTA tubes (Franklin Lakes, NJ, USA) were sent to LabCorp (Houston, TX, USA) for WBC count differential testing. 

Reactive oxygen species (ROS): ROS levels were quantified in RBC collected on D1, D15 and D30 with a fluorometric assay based on the oxidation of the fluorochrome 2,7-dichlorofluororescein-diacetate (DCFH-DA, Sigma-Aldrich, St. Louis, MO, USA), as previously reported with modifications [12]. Briefly, RBC were diluted with phosphate saline buffer (PBS) to a final concentration of 5%. RBC samples at 5% (50 µL) were pipetted into a 96-well plate followed by 50 µL of 100 µM of DCFH-DA. Relative Fluorescent Units (RFU) were monitored at 520 nm emission and 485 nm excitation after 30 min reaction at 37 °C protected from light in a FLUOstar Omega microplate reader (BMG Labtech Inc., Durham, NC, USA). Hemoglobin concentrations (mg) were determined using Drabkin’s reagent (RICCA, Arlington, TX). Briefly, diluted RBC samples (5 µL) were pipetted into a 96-well plate followed by 200 µL of Drabkin’s reagent containing Brij 35 (30% *w*/*v*). Absorbance values were read at 540 nm in a FLUOstar Omega microplate reader after 5 min reaction at 56 °C protected from light with gentle shaking. A standard curve was prepared using serial dilutions of hemoglobin (0–180 mg/mL). An average of four RFU values were normalized by hemoglobin concentration and reported as RFU/mg of hemoglobin. 

### 2.7. Blood Lipid Profile

Blood samples (approximately 10 mL) collected on D1 and D30 in Vacutainer ^®^ SST^TM^ tubes (BD, Franklin Lakes, NJ, USA) were left undisturbed for 30 min at room temperature followed by centrifugation at 2000 rpm for 10 min at 4 °C to obtain blood serum. Samples were sent to LabCorp (Houston, TX, USA) for total cholesterol, LDL-cholesterol (LDL), HDL-cholesterol (HDL), triglycerides (TG), and non-HDL cholesterol analyses. 

### 2.8. Liver Enzymes

Blood serum collected on D1 and D30 as detailed above were analyzed by LabCorp (Houston, TX, USA) for levels of alanine aminotransferase (ALT), aspartate aminotransferase (AST), and alkaline phosphatase (APh). 

### 2.9. Hemoglobin A1c (HbA1c) and Estimated Average Glucose (eAG)

HbA1c and eAG analyses were performed by LabCorp (Houston, TX, USA) on blood samples (approximately 5 mL) collected on D1 and D30 in Vacutainer^®^ EDTA tubes (Franklin Lakes, NJ, USA). 

### 2.10. Statistical Analysis

Statistical analyses were performed using Graphpad Prism, Version 9.3.1 (GraphPad Software, La Jolla, CA, USA). Data are presented as mean with 95% confidence interval (CI), unless otherwise indicated. Normality tests were performed before all analyses. Data not conforming to normal distribution based on the Shapiro–Wilk test were log-transformed and indicated accordingly. The use of medication was considered in data analysis as covariate and deemed not significant for outcomes. Differences in D1 values between cherry and placebo groups were evaluated using parametric or non-parametric unpaired t-tests. Age, gender, BMI, and HEI scores were evaluated as covariates, but their effect on the dependent variables were deemed not significant (*p* > 0.05). 

The effect of treatment, study day, and their interaction were evaluated using a mixed effect analysis or 2-way ANOVA followed by Šidák multiple comparison tests. When differences between cherry and placebo were significant at D1, the difference between D30 and D1 (Δ) was computed, and marginal means were determined considering D1 values as a covariate. Statistical differences between marginal means were determined by unpaired t-test (parametric or non-parametric). Data statistically significant (*p* ≤ 0.05) due to treatment effect was stratified by BMI (low: 30–34, high: 35–40) and gender (male, female) to perform the unpaired t-test for Δ values between subjects in cherry and placebo groups nested by BMI and gender. 

Spearman correlations analyses were performed in GraphPad Prism to determine r and *p* values for correlations between Δ values of variables that showed significance in and/or between cherry and placebo groups. Correlations with r > 0.45 and *p* ≤ 0.05 were illustrated using RStudio version 1.4.1717. 

## 3. Results

### 3.1. Participant Flow Diagram, Baseline Characteristics and Compliance

**Figure 2:**
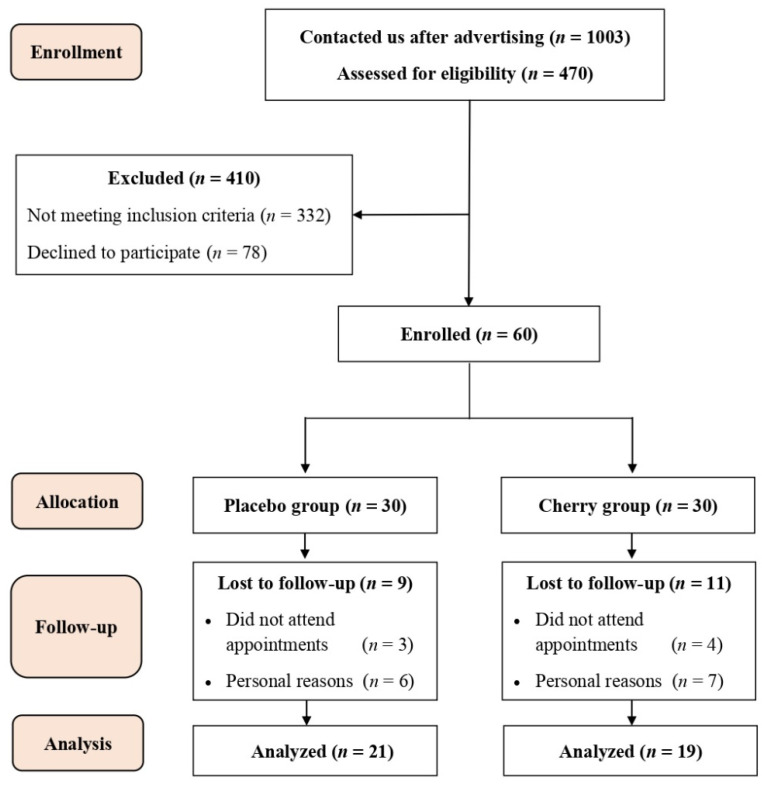
Participant flow diagram for recruitment, randomization, and data analysis.

A total of 1003 individuals responded to our online survey, and 470 participants met the eligibility criteria. Out of the 470 participants, 60 were enrolled in the study and randomly allocated into the placebo (*n* = 30) and cherry group (*n* = 30). Twenty participants (nine from placebo and 11 from cherry groups) withdrew from the study during follow-up visits due scheduling conflicts and personal reasons (Figure 2).

Upon randomization, anthropometric and physiological variables measured during screening visits showed no significant differences between placebo and cherry groups ( Demographic characteristics of all participants are shown in Supplementary Table S3. Treatment compliance was estimated as 95% for the placebo group and 91% for the cherry group. 

### 3.2. Nutritional Patterns, Anthropometric and Physiological Assessments

Nutrient patterns and HEI scores showed no significant differences between cherry and placebo groups (Supplementary Table S4). HEI scores were 40.7 and 42.6 for cherry and placebo, respectively, indicating the poor adherence to DGA. 

**Figure 3:**
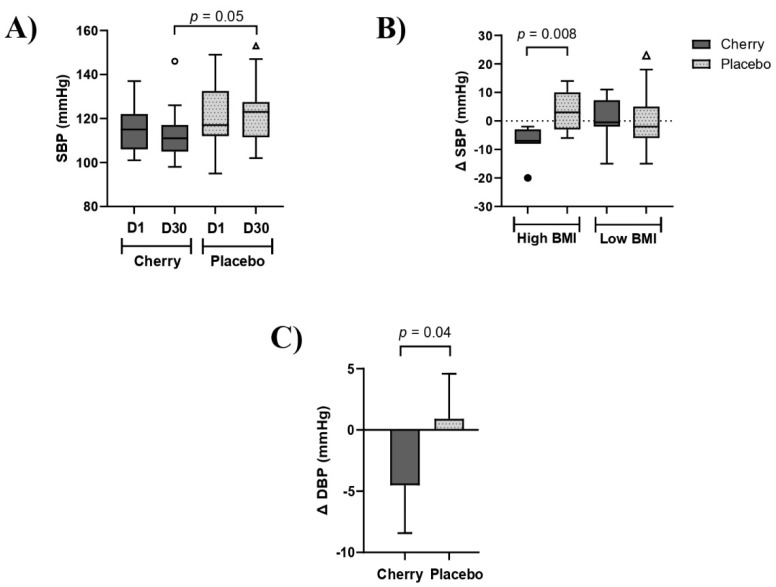
DSC supplementation decreased blood pressure in obese adults. (**A**) SBP levels in cherry and placebo groups. (**B**) Δ SBP (mmHg) stratified by BMI levels. Box plots show lower quartile, median, upper quartile, and Tukey whiskers. Data not included in the whiskers are plotted as outliers. Difference between treatments was assessed by Mann-Whitney test (**C**) Δ DBP (mmHg) levels in cherry and placebo groups. Data from DBP are estimated marginal means of Δ values (D30-D1) obtained after adjustment for significant D1 values. Difference between treatments was assessed by unpaired t-test. High BMI: 35–40. Low BMI: 30–34.

Participants allocated to the cherry group did not notice changes in health symptoms except for one participant reporting uncomfortable symptoms of gas and bloating at the end of the 30-day intervention. The anthropometric and physiological data analyses are shown in Table 1. Results showed there were no significant differences between cherry and placebo groups at D30 for anthropometric and physiological measurements. However, BW, BMI, and HR values increased significantly at D30 compared to D1 in the placebo group while no significant changes were found in the cherry group over the intervention period. SBP was significantly lower in the cherry compared to the placebo group (*p* = 0.05) (Table 1 and Figure 3A). Furthermore, changes in SBP (Δ SBP) showed that subjects with high BMI benefited from DSC intervention with Δ SBP being decreased (−7.57) significantly compared to placebo, whose Δ SBP increased (3.45) (Figure 3B and Supplementary Table S4). No significant differences between treatments were found in low-BMI subjects and when groups were stratified by gender. 

Raw data shown in Table 1 show that DBP was significantly different between cherry and placebo groups at D1, with placebo being higher than cherry at baseline (83.95 vs. 77.53, *p* = 0.05). Therefore, the Δ values (D30-D1) were computed to evaluate differences between treatments. Results showed that marginal mean and 95% CI for Δ DBP in the cherry group was −4.52 (−8.29, −0.75), and significantly lower than Δ DBP in the placebo group [0.91 (−2.66, 4.48)] (*p* = 0.04) (Figure 3C). When data was stratified by BMI, Δ DBP decreased in high-BMI cherry participants (−6.48) while an opposite trend was found in the placebo group (0.28), although the difference between groups did not reach significance (*p* = 0.13) (Supplementary Table S5). No differences were found when data was stratified by gender. 

### 3.3. Blood Biomarkers of Inflammation and Oxidative Stress

#### 3.3.1. Cytokines

Plasma levels of inflammatory cytokines are presented in Table 2. D1 levels of all inflammatory cytokines assessed in this study were not statistically different between cherry and placebo groups. IL–1RA is a cytokine that prevents the binding of IL–1 and exerts anti-inflammatory properties [18,19]. A significant effect of day (*p* = 0.01) was detected for IL–1RA with levels in the placebo group decreasing significantly at D30 compared to D1 (*p* = 0.002) (Table 2), while no significant changes were detected in the cherry group. 

IL-10 is an anti-inflammatory cytokine that exerts a key role in immune responses [20,21]. The 2-way ANOVA revealed a significant interaction of treatment and day (*p* = <0.0001) for IL-10 (Table 2). IL-10 increased significantly at D30 compared to D1 in the placebo group (*p* < 0.0001). Furthermore, IL-10 was significantly higher in placebo than in the cherry group at D30 (*p* = 0.04). 

MCP-1 is an inflammatory marker that plays a key role as a CVD risk factor [22]. Results in Table 2 showed a significant interaction between treatment and day (*p* = 0.03) for MCP-1 values. MCP-1 tended to decrease in the cherry group while the placebo group had an opposite trend without reaching significance. 

**Figure 4:**
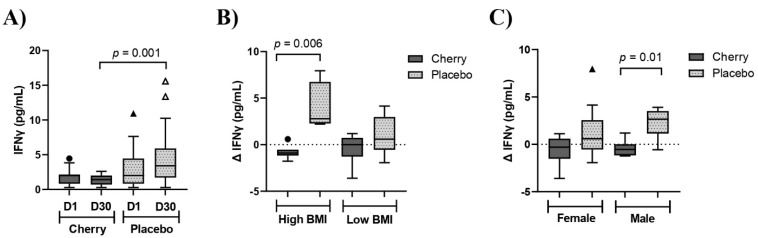
DSC supplementation decreased IFNγ in male obese adults with high BMI. (**A**) IFN**γ** (pg/mL) levels in cherry and placebo groups. Difference between treatments was determined by 2-way ANOVA analysis followed by Šídák multiple comparison test. (**B**) Δ IFN**γ** (pg/mL) stratified by low (30–34) and high (35–40) BMI (**C**) IFN**γ** (pg/mL) stratified by sex. Difference between treatments was assessed by Mann-Whitney test. Box plots show lower quartile, median, upper quartile, and Tukey whiskers. Data not included in the whiskers are plotted as outliers. Δ: D30-D1.

IFNγ is a pro-inflammatory cytokine that has been implicated in the pathogenesis of CVD [23]. A significant interaction of treatment and day (*p* = 0.01) was detected for IFNγ. Results showed that IFNγ tended to decrease in the cherry group at D30 compared to D1, while levels in the placebo group showed an opposite trend. Moreover, IFNγ was significantly lower in the cherry group than in the placebo group at D30 (*p* = 0.001) (Table 2 and Figure 4A). When Δ IFNγ was stratified by BMI, there was a significant difference between cherry and placebo subjects with high BMI (*p* = 0.006), as well as between cherry and placebo male participants (*p* = 0.01) (Figure 4B,C and Supplementary Table S6). No significant difference was found in Δ IFNγ for low-BMI and female participants.

IL-1β is a cytokine that promotes inflammation and is elevated in obese individuals [18]. IL-1β was significantly different between cherry and placebo groups at D1 (*p* = 0.05), thus, the mean change (Δ) from D30 to D1 was computed to evaluate differences between treatments. Results showed that Δ IL-1β tended to be lower in the cherry group (Δ = −0.02, 95%CI: −0.30, 0.25) compared to placebo (Δ = 0.13, 95% CI: −0.18, 0.45) but the difference between treatments did not reach significance. 

CRP is a marker of systemic inflammation that has emerged as a major cardiovascular risk factor [24]. Elevated levels of CRP are linked to metabolic syndrome, T2D, CVD and obesity [25,26]. Results from qualitative analysis of CRP levels were calculated as fold of D1 values. Data showed a significant interaction between treatment and day (*p* = 0.02) for CRP levels. CRP at D30 (fold of D1) tended to decrease in the cherry group compared to D15 (fold of D1), while an opposite trend was found for the placebo group, but changes in CRP levels did not reach significance (Table 2). 

Similarly, no significant changes in plasma IL-18, TNF-α, RANTES and IL-6 levels over the intervention period nor between cherry and placebo groups at each time point were detected (Table 2). These results strongly indicate that high-BMI subjects benefited the most from DSC consumption because of the improvements in SBP and DBP levels as well as the lowering effects on proinflammatory IFNγ, with important implications for CVD protection. 

#### 3.3.2. ESR and ROS Levels

ESR is a test used to detect inflammatory conditions [27]. Obesity and related metabolic syndromes have been associated with increased ESR [27]. Similarly, high levels of ROS have been correlated to obesity and related disorders [28]. Results showed that ESR values tended to decrease in the cherry group by D30 compared to D1, while levels in the placebo group showed an opposite trend (Table 2). In contrast to ESR trends, levels of ROS tended to decrease in both cherry and placebo groups but only placebo at D15 was significant compared to D1 (*p* = 0.03) (Table 2). 

#### 3.3.3. WBC Count

The WBC count is a common marker of inflammation [29]. Obese individuals with elevated WBC counts have a higher risk of developing T2D [29]. Results from the WBC count showed no significant changes between cherry and placebo groups (Supplementary Table S7). 

### 3.4. Cholesterol Levels

Results from the blood lipid panel showed similar levels of TC, TG, HDL-c, LDL-c, and non-HDL between cherry and placebo groups at D1 and at D30 (Table 3). In general, only TG levels increased significantly within time points in cherry (*p* = 0.006) and placebo (*p* = 0.02) groups.

### 3.5. HbA1c and eAG

Obesity is linked to impaired glucose tolerance and insulin resistance. HbA1c and eAG tests monitor glucose levels in the blood and are often used to diagnose diabetes. Results showed that Hb1Ac and eAG values were similar between cherry and placebo groups and no significant changes were found over time (Table 3). 

### 3.6. Liver Enzymes

APh, AST and ALT blood tests are used as indicators of liver health. Results showed no significant differences in APh, AST, ALT between cherry and placebo groups as well as no significant changes over time (Table 3). 

### 3.7. Correlation Analysis

**Figure 5:**
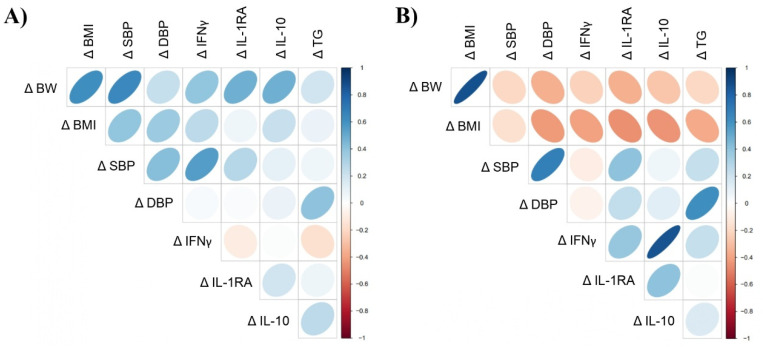
Spearman’s correlations between variables showing significant change over the intervention period in (**A**) cherry and (**B**) placebo. Direction of ellipses represents positive or negative correlations, and the width represents the strength of the correlation (narrow ellipse = stronger correlation). Δ = D30-D1. Spearman r coefficient and *p* values are shown in Supplementary Table S8.

The Spearman correlation analysis between variables showing significant change over the study period showed positive and significant correlations between Δ BW, Δ BMI, Δ SBP and Δ IL-10 in the cherry group. Likewise, Δ SBP showed significant positive correlations with Δ BMI and Δ IFNγ (Figure 5A and Supplementary Table S8). Although not significant, negative correlations between Δ BMI, Δ IFNγ, Δ IL-1RA and Δ IL-10 were detected in the placebo group (Figure 5B and Supplementary Table S8). Δ BMI showed a strong positive correlation with Δ BW. Similarly, Δ IL-1RA showed significant positive associations with Δ BW, Δ BMI and Δ SBP, while Δ IL-10 showed a strong positive correlation with Δ IFNγ. Such correlations were not detected in the cherry group. Likewise, Δ TG was positively correlated with Δ DBP, while such correlation was not detected in the cherry group despite an increase in TG levels (Figure 5B and Supplementary Table S8). 

## 4. Discussion

This study assessed the impact of DSC supplementation on inflammation, dyslipidemia, and liver function in obese adults. Results from this study showed that DSC intervention decreased SBP and DBP in obese subjects. Moreover, changes detected in SBP correlated positively with BW and BMI. Studies have shown that BMI is positively correlated with SBP and DBP, evidencing the importance of controlling anthropometric parameters that are increased in obese individuals [30]. Interestingly, participants with a high BMI seemed to benefit more from DSC blood-pressure-lowering effects. These results are relevant because obesity is associated with CVD risk factors including hypertension, thus the modulation of SBP exerted by DSC suggests its potential to improve cardiovascular health in obese adults with a high BMI. Consistent with our findings, a clinical study reported a decrease in SBP after consumption of Bing sweet cherries (280 g/day) for 28 days in healthy adults, although this effect did not attain significance [14]. Moreover, an acute decline in SBP and DBP was reported after consumption of sweet cherry juice (200 mL) over a period of 2 h [15]. Similar results were demonstrated in clinical studies conducted with tart cherry juice. For example, Chai and colleagues [31] reported a decrease of 4 mmHg in SBP in older adults who consumed tart cherry juice daily (480 mL) for 12 weeks, an effect attributed to the polyphenol content (450 mg gallic acid equivalent/mL) in tart cherry juice. DSC are also considered a rich source of polyphenols, specifically anthocyanins, which have been credited with multiple health benefits. Even though SBP and DBP were within normal levels in both cherry and placebo groups, the effect of DSC intervention in blood pressure suggests the potential of DSC to protect against hypertension and CVD risk, mainly in subjects with BMI ≥ 35. 

IL-1RA is an antagonist to the pro-inflammatory cytokine IL-1 that can attenuate inflammatory processes [19]. Likewise, IL-10 is also an anti-inflammatory cytokine that can suppress the production of pro-inflammatory cytokines. It has been proposed that circulating concentrations of IL-1RA and IL-10 are increased in obese subjects to counterbalance chronic inflammatory states associated with obesity [18,19]. Interestingly, polyphenol-rich foods have been reported to enhance the release of anti-inflammatory cytokines to regulate immunity [32]. In this study, no significant changes in plasma IL-1RA were detected after DSC supplementation. A previous study reported IL-1RA increase after 28-day DSC var. Bing consumption (280g/day) on subjects with moderately elevated CRP levels (mean: 3.5 mg/L) [14]. Moreover, it has been reported that patients with T2D and metabolic syndrome have increased IL-1RA levels, and these are positively correlated with CRP, BF (%) and weight gain [19]. This may explain why IL-1RA levels did not change significantly in the cherry group, since subjects did not present the conditions outlined above (e.g., T2D, elevated CRP). However, Δ IL-1RA was positively correlated with Δ BW in the cherry group, but did not reach significance (r = 0.041, *p* = 0.08) (Figure 5A).

Similarly, plasma IL-10 levels did not change significantly in the cherry group but were significantly lower compared to the placebo group at D30. The increase of IL-10 in the placebo group at D30 (*p* < 0.0001) supports the fact that placebo inflammatory conditions increased over the study period as a homeostatic response from the body [33]. Furthermore, this was demonstrated by the strong positive correlation between Δ IL-10 and Δ IFNγ in the placebo group (r = 0.81, *p* = 0.0001) (Figure 5B). Therefore, increased IL-10 levels in the placebo group might represent feedback from elevated pro-inflammatory cytokines upregulated in obesity, including IFNγ [30]. This was confirmed by IFNγ levels in the cherry group that were significantly lower than in the placebo group at D30 and the lack of correlation between IL-10 and IFNγ in the cherry group (Figure 5A). Since this is the first study reporting modulation of IFNγ upon DSC consumption, further research will be needed to understand whether DSC can support the inflammatory/anti-inflammatory balance in similar contexts. 

IFNγ and MCP-1 are pro-inflammatory cytokines that play an important role in obesity-induced inflammatory responses and are linked to an increased risk for CVD and type 2 diabetes [22,23]. Therefore, the suppression of IFN and MCP-1 might be considered as a relevant dietary strategy to lessen obesity-associated complications. Findings from this study showed that DSC intake for 30 days lessened IFNγ levels by ~30%, whereas in the placebo group, IFNγ levels were upregulated by ~58%. Likewise, even though changes in MCP-1 levels did not reach significance due to the high variability among subjects, a trend in MCP reduction (~5%) was observed after DSC supplementation, while in the placebo group MCP-1 levels were upregulated by ~14% over the study period. Furthermore, stratification of IFNγ data by BMI and gender confirmed the greater benefit of DSC intervention for subjects with high BMI and for male subjects (Supplementary Table S5). Future studies with obese subjects should focus on those with a BMI: 35–40 to confirm the beneficial effects of DSC in decreasing IFNγ and other biomarkers associated with IFNγ and macrophage activation. Interestingly, changes detected in IFNγ presented a positive correlation with SBP in the cherry group (r = 0.48, *p* = 0.04). Ribeiro et al. [30] also demonstrated a similar correlation in eutrophic but not in overweight women. Obesity is associated with increased metabolic risk factors, which in turn are linked to inflammatory processes. Therefore, our results suggest that DSC might reduce pro-inflammatory environment and, consequently, the risk of cardiovascular morbidities. This study is reporting for the first time lowered IFNγ in obese individuals in response to DSC supplementation. This beneficial outcome could be attributed to the polyphenols present in DSC, in particular anthocyanins. This statement is supported by a double-blind placebo-controlled trial, in which plasma IFNγ levels decreased after a twelve-week supplementation with an anthocyanin-rich acai beverage [34].

Obesity predisposes to a chronic inflammatory state which is accompanied by higher levels of pro-inflammatory cytokines such as RANTES, TNF-α, IL-6 and IL-18. Moreover, CRP and ESR are inflammatory markers, levels of which are increased under metabolic abnormalities such as obesity and related metabolic syndromes [27,35]. In this study, DSC consumption did not improve plasma levels of these inflammatory biomarkers (Table 2). Likewise, a study conducted with older individuals with mild-to-moderate dementia demonstrated that serum IL-6 was not altered after a 12-week sweet cherry juice (200 mL) consumption [36]. Similarly, Vargas et al. [37] found no significant changes in inflammatory markers such as CRP, urinary prostaglandin E2 and thromboxane B2 after sweet cherry juice supplementation (426 g/day) in individuals at risk or with metabolic alterations. However, contrasting results have been also reported. For example, Bing sweet cherries intake (280 g/day) for 28 days significantly decreased plasma concentrations of IL-18, RANTES and nitric oxide (NO) in healthy adults [13,14]. In addition, clinical trials providing tart cherry juice for four and 12 weeks observed modulatory effects on inflammatory and oxidative stress markers in middle-aged and older adults [38,39]. Discrepancies between results obtained in this study and previous research may be attributed to the baseline inflammatory status of study participants. It has been previously suggested that individuals with elevated metabolic or inflammatory conditions are more likely to benefit from anthocyanin or polyphenol interventions [40,41]. In this study, ESR values were within clinically normal ranges [42], and IL-6 and IL-18 plasma levels were similar to those reported previously in non-obese individuals [14,43] suggesting that participants displayed limited inflammatory status. Therefore, the selection of subjects with high levels of inflammatory markers should be considered as inclusion criteria for future studies aiming to assess the effects of DSC intervention on inflammation. 

Findings from this study demonstrated that anthropometric (BW and BMI) and HR values as well as IFNγ worsened in the placebo group, accompanied by the counteracting response of increased anti-inflammatory IL-10 and IL-1RA, while in the cherry group these variables remained unchanged over the DSC intervention. A positive and significant correlation between changes in SBP and IFNγ in the cherry group suggests that DSC-mediated IFNγ downregulation contributed to reduce SBP with implications for improved vascular function, as previously reported [44].

Oxidative stress is caused by the imbalance between production of ROS and defense mechanisms involving antioxidant and detoxifying enzymes in the human body. This condition leads to oxidative stress, damage of cells, inflammation, and the initiation and progression of chronic and degenerative diseases, including CVD. Dietary antioxidants such as the DSC polyphenols can protect against oxidative stress due to their ability to inhibit ROS production and remove free radicals by becoming oxidized themselves, thus preventing oxidation reactions that damage cellular macromolecules. In addition, polyphenols inhibit pro-inflammatory molecular signaling pathways which are activated by oxidative stress. Therefore, the antioxidant activity and inhibition of enzymes associated with production of pro-inflammatory molecules by polyphenols from DSC may be the underlying molecular mechanisms by which the production of proinflammatory cytokine IFNγ was suppressed, accompanied by IL-1β lowered levels.

DSC supplementation did not modulate levels of blood lipids (TC, TG, HDL, LDL, and non-HDL) in obese adults. These results are consistent with previous studies investigating the role of tart cherry on cardiometabolic risk and reporting no effect on blood lipid levels [40,45]. Likewise, a recent meta-analysis of seven randomized clinical trials showed no significant effects of tart or sweet cherry intake on serum lipids [46]. In contrast, previous studies in rodents fed high-fat diets reported the antihyperlipidemic effects of DSC [47,48], while a meta-analysis of thirty-two randomized controlled trials reported that the efficacy of anthocyanin-rich foods on cardiometabolic health were only evident in subjects with dyslipidemia [49]. Similarly, a significant decrease in oxidized LDL levels was reported in adults with metabolic syndrome (e.g., elevated TG and reduced HDL) after 12-week tart cherry juice supplementation [45]. Baseline levels of TC, HDL, and TG in this study were within normal limits (TC: 125 to 200 mg/dL, HDL: ≥40 mg/dL and TG: <150 mg/dL) [50,51], which might explain the lack of effect of DSC supplementation. Therefore, healthy levels of cholesterol in study participants before supplementation may be considered as exclusion criteria when the effects of DSC supplementation on blood lipids are pursued as a study objective. 

Regarding TG levels in cherry and placebo groups, it is important to note that such levels remained within normal limits before and after intervention. Diet and lifestyle factors are key drivers of elevated TG. The significant increase in TG levels observed in cherry and placebo groups may be related to the total fat intake exceeding 30% of the total energy intake in both groups [52] (Supplementary Table S4). In particular, the intake of saturated and trans-fats can also contribute to unhealthy weight gain and thus increased TG production, as well as a diet high in simple sugars (CHO). In this study, CHO intake represented between 45–65% of the total daily calories, which falls within recommended DGA. However, it is likely that the CHO intake might have been higher, as participants underestimated their caloric intake. Even though TG levels increased in cherry and placebo groups, a high and significant correlation between changes in TG and DBP was detected only in the placebo group (Figure 5B). This is relevant considering the association between TG and risk of atherosclerotic cardiovascular disease [53].

It is well established that obesity increases the risk of developing diabetes and liver diseases. The anti-diabetic effects of sweet and tart cherries extracts have been reported in animal models [54,55]. Our research team has previously reported improved liver function by the reduction of liver lipids and protein carbonyls in obese diabetic (db/db) mice [12]. However, these beneficial outcomes could not be translated into this human study. Results showed that HbA1c and eAG levels were similar and within normal ranges (HbA1c: 4–6%, eAG: 70–126 mg/dL) [56], in both cherry and placebo groups after intervention. Likewise, DSC or cherry products did not alter fasting glucose and insulin in healthy subjects with normal Hb1A c and eAG values, as reviewed by Kelley et al. [41]. Thus, the recruitment of subjects with HbA1c and eAG levels above normal levels should be considered in future studies when the assessment DSC in diabetic risk is considered as a specific aim. 

As noted previously, the beneficial effects of DSC consumption seemed to be dependent on the health status of participants before intervention. In this context, no significant changes were found in mean levels of liver enzymes assessed in this study, which were within clinically normal ranges (APh = 30–120 IU/L, AST = 0–35 IU/L, ALT = 0–45 IU/L) [57]. The potential role of polyphenol-rich diets in the prevention of liver diseases has been reviewed previously; [58] however, data from clinical trials are limited. For example, the consumption of fresh mangoes for 12 weeks significantly reduced AST levels in overweight and obese adults, while no effects were observed for ALT and APh [59]. Similarly, citrus limonin glucoside supplementation (500 mg/day) for 127 days decreased concentrations of ALT and APh in overweight adults [60]. Another clinical study reported significant reductions of ALT in NAFLD patients after a 12-week consumption of resveratrol (500 mg/day) [61]. These studies suggest that subjects with a high risk of developing liver disease may benefit from DSC intake. Thus, borderline and/or above normal levels of liver enzymes should be considered as a recruiting inclusion criterion to assess the effects of DSC on liver function. 

This study is the first, to our knowledge, to evaluate the effect of DSC supplementation in free-living obese adults. The randomization method, which considered the prognostic variables BMI, age, and gender, ensured the balance of most of the analyzed baseline data in both experimental groups. A statistical analysis on data from subjects on medication was performed to find out if DSC intervention could benefit them as complementary intervention for populations having a metabolic condition under treatment, but no significant effects were found mainly because of the small sample size (cherry: *n* = 3 and placebo: *n* = 3). However, the inclusion of a subset of participants with medications was not significant as a covariate.

There were no significant changes in the cherry group regarding most of the anthropometric and physiological variables, since participants did not modify their diet and physical activity during the intervention. In contrast, BW, BMI, and HR worsened in the placebo group over the intervention period. Lastly, no serious adverse effects were reported by participants in the cherry group, and compliance was ~90%. Therefore, it is reasonable to highlight the benefits of DSC whole fruit and DSC juice as a healthier alternative to artificially sweetened beverages. 

Among the limitations to be acknowledged in this study are the participant’s self-reported diet and physical activity. Participant’s MyfitnessPal reports showed fewer calories (1911 and 2269 Kcal/day for cherry and placebo groups, respectively) than expected considering age, sex, BW, and BMI (2522 and 2563 Kcal/day in cherry and placebo groups, respectively) according to the Nutribase 19 Pro software (v.19.2).

This under-reporting of energy intake affects estimates of nutrient intakes in clinical studies [62]. Participants were also encouraged to maintain their usual physical activity, but this parameter was monitored halfway through the study and not accounted for in data analysis. Moreover, this study was conducted on obese individuals with limited metabolic disturbances, commonly referred as metabolically healthy obese (MHO) [63]. Fasting insulin, plasma triglycerides, and inflammatory states are lower in MHO; hence, bioactive-rich compounds are less likely to induce benefits in this population. However, even in this cohort with MHO, biomarkers associated with cardiovascular risk such as IFNγ were lowered by DSC intervention, implying the protection exerted by DSC. Moreover, DSC intervention might contribute to vascular health through SBP and DBP modulation, an outcome that should be interpreted with caution, since it was proven only in obese populations with a BMI between 30–40. This study provides encouraging results for future clinical studies assessing DSC intake in metabolically impaired subjects with a BMI ≥ 35. Additionally, considering the possible benefits for heart risk factors, future studies should consider adding other biomarkers of heart disease risk as recruitment criteria (e.g., hypercholesterolemia and/or hypertension), as well as DSC intervention for more than 30 days. 

## 5. Conclusions

Overall, results from this study demonstrated that DSC intervention seemed to protect obese subjects from worsened obesity-related conditions such as increases in BW, BMI, and HR observed in the placebo group. Moreover, DSC consumption may attenuate inflammation and cardio-metabolic dysregulation in obese adults through the modulation of IFNγ and blood pressure. Interestingly, these outcomes seemed to be beneficial in obese subjects with a high BMI. Moreover, significant changes in anti-inflammatory IL-10 detected in the placebo group suggest an attempt to restore homeostasis to counterbalance obesity-related inflammation, while no changes were detected in the cherry group. Results from this study demonstrated the benefits of DSC for blood pressure and inflammation and suggest the intake of DSC as a dietary approach to enhance health in obese individuals who are at risk of developing chronic diseases. Findings of this study remain to be confirmed in larger intervention studies with metabolically impaired obese subjects.

## Figures and Tables

**Table 1:** Anthropometric and physiological parameters in cherry and placebo groups.

Variable	Day	Treatment		Mixed Effect Model *p* Values			Sliced by Treatment and/or Time Point
		Cherry (*n* = 19)	Placebo (*n* = 21)	Trt.	Day	Trtxday	
BW (kg)	D1	94.26 (88.37, 100.16)	93.80 (87.41, 100.18) ^X^	*0.91*	*0.003*	*0.26*	Placebo (*p* = 0.0002) ↑ D30
	D15	93.75 (87.49, 100.01) *n* = 17	94.69 (87.93, 101.45) *n* = 20				
	D30	94.71 (88.80, 100.63)	94.86 (88.14, 101.58) ^Y^				
BMI (kg/m^2^)	D1	33.55 (32.16, 34.94)	33.05 (31.87, 34.22) ^X^	0.69	0.01	0.30	Placebo (*p* = 0.006) ↑ D30
	D15	33.58 (33.89, 33.28) (*n* = 16)	33.16 (31.93, 34.39) *n* = 20				
	D30	33.68 (32.25, 35.11)	33.47 (32.29, 34.64) ^Y^				
BF (%)	D1	33.68 (30.31, 37.05)	36.03 (33.74, 38.32)	0.25	0.56	0.45	NS
	D15	34.88 (31.27, 38.49) *n* = 16	35.63 (33.38, 37.88) *n* = 20				
	D30	33.92 (30.75, 37.09)	36.07 (33.96, 38.17)				
WC (cm)	D1	102.08 (96.51, 107.65) *n* = 16	101.81 (95.6,2 108.00) *n* = 17	0.83	0.68	0.46	NS
	D15	101.86 (96.66, 107.07) *n* = 17	100.54 (95.86, 105.21) *n* = 19				
	D30	101.47 (96.38, 106.56) *n* = 19	101.76 (96.35, 107.17) *n* = 21				
SBP (mmHg)	D1	115.32 (110.20, 120.43)	121.43 (114.82, 128.04)	0.03	0.23	0.49	Cherry vs. Placebo D30 (*p* = 0.05)
	D15	112.00 (104.18, 119.82) *n* = 16	120.20 (115.19, 125.21) *n* = 20				
	D30	112.84 (107.57, 118.11) ^A^	122.33 (116.47, 128.20) ^B^				
DBP (mmHg)	D1	77.53 (73.40, 81.65) ^A^	83.95 (79.88, 88.02) ^B^	0.007	0.32	0.26	Cherry vs. Placebo D1 (*p* = 0.05) Cherry vs. Placebo D30 (*p* = 0.005)
	D15	77.31 (72.26, 82.36) *n* = 16	81.50 (78.28, 84.72) *n* = 20				
	D30	74.63 (70.60, 78.66) ^A^	83.38 (79.07, 87.69) ^B^				
HR (bpm)	D1	73.05 (65.45, 80.66)	73.00 (69.36, 76.64)	0.80	0.03	0.52	Placebo (*p* = 0.04) ↑ D30
	D15	71.50 (66.04, 76.96) *n* = 16	72.42 (68.09, 76.75) ^X^ *n* = 19				
	D30	74.79 (68.58, 81.00)	77.52 (72.81, 82.24) ^Y^				
OS (SpO2%)	D1	97.05 (96.11, 98.00)	97.38 (96.78, 97.98)	*0.77*	*0.99*	*0.06*	NS
	D15	97.33 (96.14, 98.53) *n* = 15	97.42 (96.93, 95.00) *n* = 19				
	D30	97.58 (97.12, 98.04)	96.62 (95.00, 98.24)				

Data are mean and 95% confidence interval (CI). Different letters indicate significant difference between treatment ^(A, B)^ and day ^(X, Y)^. Data was analyzed by fitting a mixed effect model, followed by Šídák multiple comparison test. Italicized *p*-values were obtained from log-transformed data. BW: body weight. BMI: body mass index. BF: body fat. WC: waist circumference. SBP: systolic blood pressure. DBP: diastolic blood pressure. HR: heart rate. OS: oxygen saturation. D1: day 1. D30: day 30. NS: nonsignificant. Differences were considered statistically significant when *p* ≤ 0.05. Trt: treatment. Trtxday: interaction treatment and day. Italics indicate that *p*-values were obtained from log-transformed data. ↑ increase.

**Table 2:** Biomarkers of inflammation and oxidative stress.

Variable	Day	Treatment		2-Way ANOVA *p* Values			Sliced by Treatment and/or Time Point
		Cherry (*n* = 19)	Placebo (*n* = 21)	Trt.	Day	Trtxday	
Inflammatory cytokines							
IL–1RA (pg/mL)	D1	10.18 (3.27, 17.09) *n* = 18	11.09 (8.15, 14.03) ^X^ *n* = 20	*0.72*	*0.01*	*0.10*	Placebo (*p* = 0.002) ↓ D30
	D15	10.55 (3.92, 17.18) *n* = 14	8.22 (5.55, 10.88) *n* = 16				
	D30	8.51 (3.81, 13.21) *n* = 18	7.00 (5.04, 8.97) ^Y^ *n* = 20				
IL–18 (pg/mL)	D1	29.58 (20.12, 39.03) *n* = 19	28.81 (17.12, 40.49)	*0.45*	*0.13*	*0.20*	NS
	D15	25.77 (16.90, 34.64) *n* = 16	18.09 (13.24, 22.93) *n* = 16				
	D30	29.40 (19.83, 38.96) *n* = 18	23.44 (15.77, 31.12)				
TNF–α (pg/mL)	D1	21.96 (15.88, 28.05) *n* = 19	29.00 (21.88, 36.11)	*0.84*	*0.40*	*0.11*	NS
	D15	28.35 (20.87, 35.82) *n* = 17	24.07 (18.18, 29.96) *n* = 18				
	D30	23.17 (17.29, 29.05) *n* = 18	22.94 (17.32, 28.57)				
RANTES (ng/mL)	D1	82.14 (39.08, 125.20) *n* = 15	97.76 (62.13, 133.39) *n* = 15	*0.69*	*0.63*	*0.77*	NS
	D15	98.59 (52.15, 145.03) *n* = 13	94.51 (42.33, 146.70) *n* = 12				
	D30	76.10 (48.66, 103.55) *n* = 15	87.77 (60.04, 115.50) *n* = 15				
IL-6 (pg/mL)	D1	9.70 (1.76, 17.65) *n* = 14	35.55 (−3.03, 74.15) *n* = 15	*0.82*	*0.32*	*0.50*	NS
	D15	7.12 (0.13, 14.12) *n* = 12	39.11 (−4.19, 82.42) *n* = 13				
	D30	7.08 (1.75, 12.42) *n* = 14	31.87 (2.80, 66.53) *n* = 15				
IL-10 (pg/mL)	D1	9.41 (6.52, 12.31) *n* = 17	9.07 (6.28, 11.86) ^X^	*0.31*	*0.0005*	*<0.0001*	Cherry vs. Placebo D30 (*p* = 0.04) Placebo (*p* = <0.0001) ↑ D30
	D30	8.62 (6.36, 10.87) ^A^ *n* = 17	15.25 (10.97, 19.52) ^B,Y^				
MCP-1 (pg/mL)	D1	297.11 (254.83, 339.39) *n* = 18	261.04 (221.94, 300.15)	0.53	0.43	0.03	NS
	D30	282.06 (237.62, 326.52) *n* = 18	298.99 (238.69, 359.29)				
IFNγ (pg/mL)	D1	1.80 (1.22, 2.38) *n* = 17	3.00 (1.49, 4.76) *n* = 15	0.01	0.68	0.01	Cherry vs. Placebo D30 (*p* = 0.001)
	D30	1.31 (0.89, 1.73) ^A^ *n* = 17	4.76 (2.48, 7.03) ^B^ *n* = 15				
IL-1β (pg/mL)	D1	0.69 (0.43, 0.94) ^A^ n = 18	2.02 (0.83, 3.22) ^B^ *n* = 14	0.02	0.15	0.75	Cherry vs. Placebo D1 (*p* = 0.05) D30 (*p* = 0.03)
	D30	0.55 (0.34, 0.76) ^A^ *n* = 18	2.30 (0.77, 3.83) ^B^ *n* = 14				
Inflammatory markers							
ESR (mm/hr)	D1	11.8 (6.7, 16.8) *n* = 18	11.5 (6.9, 16.1) *n* = 20	*0.97*	*0.71*	*0.93*	NS
	D30	10.7 (7.9, 13.5) *n* = 18	12.3 (9.6, 14.9) *n* = 20				
CRP (*)	D15	2.27 (1.38, 3.16) *n* = 14	1.51 (0.85, 2.17) *n* = 13	0.65	0.45	0.02	NS
	D30	1.61 (1.00, 2.23) *n* = 17	2.80 (1.40, 4.20) *n* = 15				
Oxidative stress							
ROS/Hb (RFU/mg)	D1	1.21 × 10^5^ (0.78, 1.32 × 10^5^) *n* = 18	1.27 × 10^5^ (0.83, 1.70 × 10^5^) ^X^	*0.72*	*0.04*	*0.43*	Placebo (*p* = 0.03) ↓ D15
	D15	1.08 × 10^5^ (0.83, 1.32 × 10^5^) *n* = 16	0.85 × 10^5^ (0.62, 1.09 × 10^5^) ^Y^ *n* = 18				
	D30	1.01 × 10^5^ (0.77, 1.24 × 10^5^) *n* = 18	0.96 × 10^5^ (0.75, 1.18 × 10^5^)				

Data are mean and 95% confidence interval (CI). Different letters indicate significant difference between treatment ^(A, B)^ and day (^X, Y)^. Data was analyzed by fitting a mixed-effect model followed by Šídák multiple comparison test. Italicized *p*-values were obtained from log-transformed data. IL-1RA: interleukin 1 receptor antagonist. IL-18: interleukin 18. TNF-α: tumor necrosis factor alpha. RANTES: regulated upon activation normal T-cell expressed and secreted. IL-6: interleukin 6. IL-10: interleukin 10. MCP-1: monocyte chemoattractant protein 1. IFNγ: interferon gamma. IL-1β: interleukin 1β. ESR: erythrocyte sedimentation rate. CRP: C-reactive protein. (*) Fold of D1 is presented for D15 and D30. ROS/Hb: reactive oxygen species/hemoglobin. D1: day 1. D15: day 15. D30: day 30. NS: nonsignificant. Differences were considered significant when *p* ≤ 0.05. Trt. = treatment. Trtxday = interaction treatment and day. Italics indicate that *p*-values were obtained from log-transformed data. ↓ decrease; ↑ increase.

**Table 3:** Biomarkers of lipid profile, glucose levels and liver enzymes.

Variable	Day	Treatment		2-Way ANOVA *p* Values			Sliced by Treatment and/or Time Point
		Cherry (*n* = 19)	Placebo (*n* = 21)	Trt.	Day	Trtxday	
Lipid profile							
TC (mg/dL)	D1	180.12 (168.35, 191.88) *n* = 17	177.65 (161.66, 193.64) *n* = 20	0.79	0.08	0.93	NS
	D30	184.41 (172.23, 196.59) *n* = 17	181.55 (163.48, 199.62) *n* = 20				
TG (mg/dL)	D1	97.47 (72.20, 122.73) ^X^ *n* = 17	110.00 (86.74, 133.26) ^X^ *n* = 20	*0.56*	*0.0003*	*0.56*	Cherry (*p* = 0.006) ↑ D30 Placebo (*p* = 0.02) ↑ D30
	D30	123.59 (87.82, 159.35) ^Y^ *n* = 17	131.20 (99.78, 162.62) ^Y^ *n* = 20				
HDL (mg/dL)	D1	46.24 (40.10, 52.37) *n* = 17	48.70 (44.40, 53.00) *n* = 20	0.54	0.51	0.67	NS
	D30	46.00 (39.70, 52.30) *n* = 17	47.60 (43.43, 51.77) *n* = 20				
LDL (mg/dL)	D1	114.71 (102.93, 126.48) *n* = 17	108.80 (95.67, 121.92) *n* = 20	0.48	0.47	0.99	NS
	D30	116.00 (104.25, 127.75) *n* = 17	110.05 (96.86, 123.24) *n* = 20				
Non-HDL (mg/dL)	D1	132.71 (118.72, 146.69) *n* = 17	128.95 (113.73, 114.17) *n* = 20	0.68	0.01	0.85	NS
	D30	138.41 (124.67, 152.16) *n* = 17	135.95 (116.95, 150.95) *n* = 20				
Hemoglobin A1c and glucose							
HbA1c (%)	D1	5.40 (5.22, 5.58) *n* = 18	5.52 (5.30, 5.75)	0.39	0.12	0.79	NS
	D30	5.43 (5.26, 5.61) *n* = 18	5.55 (5.33, 5.77)				
eAG (mg/dL)	D1	108.44 (103.21, 113.68) *n* = 18	112.00 (105.49, 118.51)	0.39	0.13	0.79	NS
	D30	109.39 (104.41, 114.37) *n* = 18	112.67 (106.37, 118.97)				
Liver enzymes							
APh (IU/L)	D1	71.07 (63.93, 78.22) *n* = 14	83.13 (67.84, 98.41) *n* = 16	0.13	0.41	0.43	NS
	D30	71.14 (63.08, 79.21) *n* = 14	85.63 (68.50, 102.70) *n* = 16				
AST (IU/L)	D1	23.53 (16.83, 30.23) *n* = 17	19.95 (17.97, 21.92) *n* = 19	*0.28*	*0.95*	*0.58*	NS
	D30	23.82 (17.17, 30.48) *n* = 17	19.58 (17.57, 21.59) *n* = 19				
ALT (IU/L)	D1	25.06 (16.19, 33.93) *n* = 16	20.05 (16.18, 23.92) *n* = 19	*0.20*	*0.72*	*0.28*	NS
	D30	26.06 (17.35, 34.77) *n* = 16	19.32 (15.57, 23.06) *n* = 19				

Data are mean and 95% confidence interval (CI). Different letters indicate significant difference between treatment ^(A, B)^ and day ^(X, Y)^. Data was analyzed by fitting a 2-way ANOVA analysis, followed by Šídák multiple comparison test. Italicized *p*-values were obtained from log-transformed data. TC: total cholesterol. TG: triglycerides. HDL: high density lipoprotein cholesterol. LDL: low-density lipoprotein cholesterol. HbA1c: hemoglobin A1c. eAG: estimated average glucose. APh: alkaline phosphatase. AST: aspartate aminotransferase. ALT: alanine aminotransferase. D1: day 1. D30: day 30. NS: nonsignificant. Differences were considered significant when *p* ≤ 0.05. Trt. = treatment. Trtxday = interaction treatment and day. Italics indicate that *p*-values were obtained from log-transformed data. ↑ increase.

## Data Availability

The datasets presented in this study are not readily available because participants did not provide consent for their data to be shared publicly. Requests to access the datasets should be directed to the corresponding author.

## Supplementary Materials

The following supporting information can be downloaded at: https://www.mdpi.com/article/10.3390/nu15030681/s1, Table S1: Nutritional and physicochemical characteristics of DSC concentrated juice, DSC powder, and reconstituted DSC drink [64,65,66]; Table S2: Formulation of placebo concentrated juice and nutritional and physicochemical characteristics of placebo concentrated drink; Table S3: Demographic characteristics of study participants; Table S4: Nutritional patterns and HEI scores in cherry and placebo groups; Table S5: Analysis of Δ SBP and Δ DBP by BMI between cherry and placebo groups; Table S6: Analysis of Δ IFNγ by BMI and gender between cherry and placebo groups; Table S7: Differential WBC count in cherry and placebo groups; Table S8: Correlation matrix between variables showing significant changes in cherry and placebo groups.

## References

[1] Blüher M. (2019). Obesity: Global epidemiology and pathogenesis. Nat. Rev. Endocrinol..

[2] Centers for Disease Control and Prevention Nutrition, Physical Activity, and Obesity. Data, Trend and Maps. https://www.cdc.gov/nccdphp/dnpao/data-trends-maps/index.html.

[3] Andersen C.J., Murphy K.E., Fernandez M.L. (2016). Impact of Obesity and Metabolic Syndrome on Immunity. Adv. Nutr..

[4] Marseglia L., Manti S., D’Angelo G., Nicotera A.G., Parisi E., Di Rosa G., Gitto E., Arrigo T. (2014). Oxidative Stress in Obesity: A Critical Component in Human Diseases. Int. J. Mol. Sci..

[5] Calder P.C., Ahluwalia N., Brouns F., Buetler T., Clement K., Cunningham K., Esposito K., Jönsson L.S., Kolb H., Lansink M. (2011). Dietary factors and low-grade inflammation in relation to overweight and obesity. Br. J. Nutr..

[6] de Heredia F.P., Gómez-Martínez S., Marcos A. (2012). Obesity, inflammation and the immune system. Proc. Nutr. Soc..

[7] Sun B., Karin M. (2012). Obesity, inflammation, and liver cancer. J. Hepatol..

[8] Polyzos S.A., Kountouras J., Mantzoros C.S. (2019). Obesity and nonalcoholic fatty liver disease: From pathophysiology to therapeutics. Metabolism.

[9] Ramírez-Moreno E., Arias-Rico J., Jiménez-Sánchez R.C., Estrada-Luna D., Jiménez-Osorio A.S., Zafra-Rojas Q.Y., Ariza-Ortega J.A., Flores-Chávez O.R., Morales-Castillejos L., Sandoval-Gallegos E.M. (2022). Role of Bioactive Compounds in Obesity: Metabolic Mechanism Focused on Inflammation. Foods.

[10] Fonseca L.R., Silva G.R., Luís Â., Cardoso H.J., Correia S., Vaz C.V., Duarte A.P., Socorro S. (2021). Sweet cherries as anti-cancer agents: From bioactive compounds to function. Molecules.

[11] Wu T., Tang Q., Yu Z., Gao Z., Hu H., Chen W., Zheng X., Yu T. (2014). Inhibitory effects of sweet cherry anthocyanins on the obesity development in C57BL/6 mice. Int. J. Food Sci. Nutr..

[12] Noratto G.D., Lage N.N., Chew B.P., Mertens-Talcott S.U., Talcott S.T., Pedrosa M.L. (2018). Non-anthocyanin phenolics in cherry (Prunus avium L.) modulate IL-6, liver lipids and expression of PPARδ and LXRs in obese diabetic (db/db) mice. Food Chem..

[13] Kelley D.S., Rasooly R., Jacob R.A., Kader A.A., Mackey B.E. (2006). Consumption of Bing Sweet Cherries Lowers Circulating Concentrations of Inflammation Markers in Healthy Men and Women. J. Nutr..

[14] Kelley D.S., Adkins Y., Reddy A., Woodhouse L.R., Mackey B.E., Erickson K.L. (2013). Sweet bing cherries lower circulating concentrations of markers for chronic inflammatory diseases in healthy humans. J. Nutr..

[15] Kent K., Charlton K.E., Jenner A., Roodenrys S. (2016). Acute reduction in blood pressure following consumption of anthocyanin-rich cherry juice may be dose-interval dependant: A pilot cross-over study. Int. J. Food Sci. Nutr..

[16] Lim C.-Y., In J. (2019). Randomization in clinical studies. Korean J. Anesthesiol..

[17] Krebs-Smith S.M., Pannucci T.E., Subar A.F., Kirkpatrick S.I., Lerman J.L., Tooze J.A., Wilson M.M., Reedy J. (2018). Update of the Healthy Eating Index: HEI-2015. J. Acad. Nutr. Diet..

[18] Ghanbari M., Momen Maragheh S., Aghazadeh A., Mehrjuyan S.R., Hussen B.M., Abdoli Shadbad M., Dastmalchi N., Safaralizadeh R. (2021). Interleukin-1 in Obesity-Related Low-Grade Inflammation: From Molecular Mechanisms to Therapeutic Strategies. Int. Immunopharmacol..

[19] Frühbeck G., Catalán V., Ramírez B., Valentí V., Becerril S., Rodríguez A., Moncada R., Baixauli J., Silva C., Escalada J. (2022). Serum Levels of IL-1 RA Increase with Obesity and Type 2 Diabetes in Relation to Adipose Tissue Dysfunction and are Reduced After Bariatric Surgery in Parallel to Adiposity. J. Inflamm. Res..

[20] Esposito K., Pontillo A., Giugliano F., Giugliano G., Marfella R., Nicoletti G., Giugliano D. (2003). Association of Low Interleukin-10 Levels with the Metabolic Syndrome in Obese Women. J. Clin. Endocrinol. Metab..

[21] Leon-Cabrera S., Arana-Lechuga Y., Esqueda-León E., Terán-Pérez G., Gonzalez-Chavez A., Escobedo G., Moctezuma J.V. (2015). Reduced Systemic Levels of IL-10 Are Associated with the Severity of Obstructive Sleep Apnea and Insulin Resistance in Morbidly Obese Humans. Mediat. Inflamm..

[22] Panee J. (2012). Monocyte Chemoattractant Protein 1 (MCP-1) in obesity and diabetes. Cytokine.

[23] Elyasi A., Voloshyna I., Ahmed S., Kasselman L.J., Behbodikhah J., De Leon J., Reiss A.B. (2020). The role of interferon-γ in cardiovascular disease: An update. Inflamm. Res..

[24] Castro A.R., Silva S.O., Soares S.C. (2018). The Use of High Sensitivity C-Reactive Protein in Cardiovascular Disease Detection. J. Pharm. Pharm. Sci..

[25] Choi J., Joseph L., Pilote L. (2013). Obesity and C-reactive protein in various populations: A systematic review and meta-analysis. Obes. Rev..

[26] Luan Y.-Y., Yao Y.-M. (2018). The clinical significance and potential role of C-reactive protein in chronic inflammatory and neuro-degenerative diseases. Front. Immunol..

[27] Alende-Castro V., Alonso-Sampedro M., Vazquez-Temprano N., Tuñez C., Rey D., García-Iglesias C., Sopeña B., Gude F., Gonzalez-Quintela A. (2019). Factors influencing erythrocyte sedimentation rate in adults: New evidence for an old test. Medicine.

[28] McMurray F., Patten D.A., Harper M.E. (2016). Reactive oxygen species and oxidative stress in obesity—Recent findings and empirical approaches. Obesity.

[29] Gu Y., Hu K., Huang Y., Zhang Q., Liu L., Meng G., Wu H., Xia Y., Bao X., Shi H. (2018). White blood cells count as an indicator to identify whether obesity leads to increased risk of type 2 diabetes. Diabetes Res. Clin. Pract..

[30] Ribeiro I.S., Pereira Í.S., Santos D.P., Lopes D.N., Prado A.O., Calado S.P., Gonçalves C.V., Galantini M.P., Muniz I.P., Santos G.S. (2019). Association between body composition and inflammation: A central role of IL-17 and IL-10 in diabetic and hypertensive elderly women. Exp. Gerontol..

[31] Chai S.C., Davis K., Wright R.S., Kuczmarski M.F., Zhang Z. (2018). Impact of tart cherry juice on systolic blood pressure and low-density lipoprotein cholesterol in older adults: A randomized controlled trial. Food Funct..

[32] Yahfoufi N., Alsadi N., Jambi M., Matar C. (2018). The Immunomodulatory and Anti-Inflammatory Role of Polyphenols. Nutrients.

[33] Ouyang W., Rutz S., Crellin N.K., Valdez P.A., Hymowitz S.G. (2011). Regulation and Functions of the IL-10 Family of Cytokines in Inflammation and Disease. Annu. Rev. Immunol..

[34] Kim H., Simbo S.Y., Fang C., McAlister L., Roque A., Banerjee N., Talcott S.T., Zhao H., Kreider R.B., Mertens-Talcott S.U. (2018). Açaí (Euterpe oleracea Mart.) beverage consumption improves biomarkers for inflammation but not glucose-or lipid-metabolism in individuals with metabolic syndrome in a randomized, double-blinded, placebo-controlled clinical trial. Food Funct..

[35] Ebrahimi M., Heidari-Bakavoli A.R., Shoeibi S., Mirhafez S.R., Moohebati M., Esmaily H., Ghazavi H., Karimian M.S., Parizadeh S.M.R., Mohammadi M. (2016). Association of Serum hs-CRP Levels with the Presence of Obesity, Diabetes Mellitus, and Other Cardiovascular Risk Factors. J. Clin. Lab. Anal..

[36] Kent K., Charlton K., Roodenrys S., Batterham M., Potter J., Traynor V., Gilbert H., Morgan O., Richards R. (2017). Consumption of anthocyanin-rich cherry juice for 12 weeks improves memory and cognition in older adults with mild-to-moderate dementia. Eur. J. Nutr..

[37] Vargas A.J., McDonnell L.N., Liu Z., Wertheim B.C., Thomson C.A., Thompson P.A. (2014). A pilot sweet cherry feeding study in overweight men: Tolerance, safety, and anthocyanin exposure. J. Funct. Foods.

[38] Chai S.C., Davis K., Zhang Z., Zha L., Kirschner K.F. (2019). Effects of Tart Cherry Juice on Biomarkers of Inflammation and Oxidative Stress in Older Adults. Nutrients.

[39] Martin K.R., Burrell L., Bopp J. (2018). Authentic tart cherry juice reduces markers of inflammation in overweight and obese subjects: A randomized, crossover pilot study. Food Funct..

[40] Kimble R., Keane K.M., Lodge J.K., Howatson G. (2021). The influence of tart cherry (Prunus cerasus, cv montmorency) concentrate supplementation for 3 months on cardiometabolic risk factors in middle-aged adults: A randomised, placebo-controlled trial. Nutrients.

[41] Kelley D.S., Adkins Y., Laugero K.D. (2018). A Review of the Health Benefits of Cherries. Nutrients.

[42] Tishkowski K., Gupta V. Erythrocyte Sedimentation Rate. https://www.ncbi.nlm.nih.gov/books/NBK557485/.

[43] El-Mikkawy D.M.E., El-Sadek M.A., El-Badawy M.A., Samaha D. (2020). Circulating level of interleukin-6 in relation to body mass indices and lipid profile in Egyptian adults with overweight and obesity. Egypt. Rheumatol. Rehabil..

[44] Travis O.K., Tardo G.A., Giachelli C., Siddiq S., Nguyen H.T., Crosby M.T., Johnson T.D., Brown A.K., Booz G.W., Smith A.N. (2021). Interferon γ neutralization reduces blood pressure, uterine artery resistance index, and placental oxidative stress in placental ischemic rats. Am. J. Physiol. Regul. Integr. Comp. Physiol..

[45] Johnson S.A., Navaei N., Pourafshar S., Jaime S.J., Akhavan N.S., Alvarez-Alvarado S., Proaño G.V., Litwin N.S., Clark E.A., Foley E.M. (2020). Effects of montmorency Tart Cherry juice consumption on cardiometabolic biomarkers in adults with metabolic syndrome: A randomized controlled pilot trial. J. Med. Food.

[46] Eslami O., Khorramrouz F., Fatahi S., Sohouli M.H., Shidfar F. (2021). A systematic review and meta-analysis of cherry (Prunus spp.) consumption on glycemic markers and lipid profile. Obes. Med..

[47] Song H., Wu T., Xu D., Chu Q., Lin D., Zheng X. (2016). Dietary sweet cherry anthocyanins attenuates diet-induced hepatic steatosis by improving hepatic lipid metabolism in mice. Nutrition.

[48] Dziadek K., Kopeć A., Piątkowska E. (2019). Intake of fruit and leaves of sweet cherry beneficially affects lipid metabolism, oxidative stress and inflammation in Wistar rats fed with high fat-cholesterol diet. J. Funct. Foods.

[49] Yang L., Ling W., Du Z., Chen Y., Li D., Deng S., Liu Z., Yang L. (2017). Effects of Anthocyanins on Cardiometabolic Health: A Systematic Review and Meta-Analysis of Randomized Controlled Trials. Adv. Nutr..

[50] MedlinePlus (Internet) Bethesda (MD): National Library of Medicine (US). Cholesterol Levels: What You Need to Know. https://medlineplus.gov/cholesterollevelswhatyouneedtoknow.html.

[51] MedlinePlus (Internet) Bethesda (MD): National Library of Medicine. Triglycerides. https://medlineplus.gov/triglycerides.html.

[52] World Health Organization (WHO) (2020). Healthy Diet: Key Facts. https://www.who.int/news-room/fact-sheets/detail/healthy-diet.

[53] Farnier M., Zeller M., Masson D., Cottin Y. (2021). Triglycerides and risk of atherosclerotic cardiovascular disease: An update. Arch. Cardiovasc. Dis..

[54] Lachin T. (2014). Effect of antioxidant extract from cherries on diabetes. Recent Patents Endocrine, Metab. Immune Drug Discov..

[55] Saleh F.A., El-Darra N., Raafat K. (2017). Hypoglycemic effects of Prunus cerasus L. pulp and seed extracts on Alloxan-Induced Diabetic Mice with histopathological evaluation. Biomed. Pharmacother..

[56] MedlinePlus (Internet) Bethesda (MD): National Library of Medicine. Estimated Average Glucose (eAG). https://medlineplus.gov/ency/patientinstructions/000966.htm.

[57] Lala V., Zubair M., Minter D.A. Liver Function Tests. https://www.ncbi.nlm.nih.gov/books/NBK482489/.

[58] Abenavoli L., Larussa T., Corea A., Procopio A., Boccuto L., Dallio M., Federico A., Luzza F. (2021). Dietary Polyphenolsand Non-Alcoholic Fatty Liver Disease. Nutrients.

[59] Rosas M., Pinneo S., O’Mealy C., Tsang M., Liu C., Kern M., Hooshmand S., Hong M.Y. (2022). Effects of fresh mango consumption on cardiometabolic risk factors in overweight and obese adults. Nutr. Metab. Cardiovasc. Dis..

[60] Kelley D.S., Adkins Y.C., Zunino S.J., Woodhouse L.R., Bonnel E.L., Breksa III A.P., Manners G.D., Mackey B.E. (2015). Citrus limonin glucoside supplementation decreased biomarkers of liver disease and inflammation in overweight human adults. J. Funct. Foods.

[61] Faghihzadeh F., Adibi P., Hekmatdoost A. (2015). The effects of resveratrol supplementation on cardiovascular risk factors in patients with non-alcoholic fatty liver disease: A randomised, double-blind, placebo-controlled study. Br. J. Nutr..

[62] Mirmiran P., Esmaillzadeh A., Azizi F. (2006). Under-reporting of energy intake affects estimates of nutrient intakes. Asia Pac. J. Clin. Nutr..

[63] Cӑtoi A.F., Pârvu A.E., Andreicuț A.D., Mironiuc A., Crӑciun A., Cӑtoi C., Pop I.D. (2018). Metabolically Healthy versus Unhealthy Morbidly Obese: Chronic Inflammation, Nitro-Oxidative Stress, and Insulin Resistance. Nutrients.

[64] Abderrahim F., Huanatico E., Repo-Carrasco-Valencia R., Arribas S.M., Gonzalez M.C., Condezo-Hoyos L. (2012). Effect of germination on total phenolic compounds, total antioxidant capacity, Maillard reaction products and oxidative stress markers in canihua (*Chenopodium pallidicaule*). J. Cereal Sci..

[65] Lage N.N., Layosa M.A.A., Arbizu S., Chew B.P., Pedrosa M.L., Mertens-Talcott S., Talcott S., Noratto G.D. (2020). Dark sweet cherry (*Prunus avium*) phenolics enriched in anthocyanins exhibit enhanced activity against the most aggressive breast cancer subtypes without toxicity to normal breast cells. J. Funct. Foods.

[66] Giusti M.M., Wrolstad R.E. (2001). Characterization and Measurement of Anthocyanins by UV-Visible Spectroscopy. Curr. Protoc. Food Anal. Chem..

