# Supplementary material for: Dark Sweet Cherry (Prunus avium) Supplementation Reduced Blood Pressure and Pro-Inflammatory Interferon Gamma (IFNγ) in Obese Adults without Affecting Lipid Profile, Glucose Levels and Liver Enzymes
Source: Nutrients. 2023 Jan 29;15(3):681. doi: 10.3390/nu15030681 (PMC9920461; doi:10.3390/nu15030681)
Supplement: Supplementary file 1 [file nutrients-15-00681-s001.zip › nutrients-2166184-supplementary.pdf]

Supplementary Table S1. Nutritional and physicochemical characteristics of DSC concentrated juice, DSC powder, and reconstituted DSC drink

**DSC concentrated juice**		
	**Range**	**Method**
**⁰Brix[a]**	68.0° ± 1.0°	AOAC 932.13
**Titratable Acidity (wt/wt as malic) [a]**	1.5 – 3.2	AOAC 942.15
**pH @ 20.0 Brix[a]**	3.2 – 4.2	pH Meter
**Color @ 520nm as absorbance[a]**	7.0 Minimum	Spectrophotometer
**Color ratio (Color @520nm / 430nm) [a]**	1.5 Minimum	Spectrophotometer
**Turbidity (NTU @ 20.0 Brix) [a]**	<100	Turbidimeter
**Total phenolics** (mg gallic acid equivalent (GAE)/mL)	7.64 ±1.67	Folin-Ciocalteu colorimetric micro-method [1]
**Anthocyanins** (mg cyanidin 3-glucoside equivalent (C3G Eq.)/mL)	1.39 ± 0.26	HPLC-MS/MS [2]
**DSC powder**		
**Color [b]**	Light to dark burgundy	Sensory
**Odor [b]**	Mild cherry, no off notes	Sensory
**Flavor [b]**	Clean cherry, no off notes	Sensory
**Total Plate Count [b]**	< 1,000 cfu/g	AOAC
**Yeast & Mold [b]**	< 10 cfu/g	FDA BAM
**Coliforms [b]**	< 3 cfu/g	AOAC
**L monocytogenes[b]**	None detected	AOAC
**Salmonella[b]**	None detected	AOAC
**Ash [c]**	21.39%	AOAC 923.03
**Calories [c]**	303 calories/100g	21 CFR Part 101
**Total carbohydrates [c]**	68.54 %	21 CFR Part 101
**Fat [c]**	1.02%	AACC 30-10/AOAC 922.06
**Moisture [c]**	4.26%	AOAC 925.10
**Protein [c]**	4.79%	Kjeldahl method
**Total Dietary Fibers [c]**	3.74%	AOAC 991.43/AACC 32.07
**Total phenolics** (mg gallic acid equivalent (GAE)/g)	3.05 ± 0.07	Folin-Ciocalteu colorimetric micro-method [1]
**Anthocyanins** (mg C3G Eq./g)	0.24 ± 0.04	pH-differential spectrophotometric method [3]
**Reconstituted DSC drink (200 mL) [d]**		
**⁰Brix**	18.05 ± 1.04	
**pH**	3.88 ± 0.04	
**Titratable Acidity** (%, as malic acid)	2.54 ± 0.10	
**Energy** (kCal)	~146	
**Dietary fiber** (g)	0.11	
**Phenolic acids** (mg gallic acid equivalent)	439.6 ± 6.6	
**Anthocyanins** (mg C3G)	70.21 ± 0.12	

[a] Data provided by *FruitSmart* in datasheet product specification. [b] Data provided by Anderson Advanced Ingredients in datasheet product specification. [c] Cherry powder analysis report provided by Analytical Food

Laboratories (AFL) (Grand Prairie, TX, USA). [d] The reconstituted DSC drink contained 50 mL DSC concentrated juice, 3g DSC powder and 150 mL water. Data are presented as mean ± SEM.

Supplementary Table S2. Formulation of placebo concentrated juice and nutritional and physicochemical characteristics of placebo concentrated drink

**Placebo concentrated drink formula [a]**	
**Ingredient**	**g/L**
Maltodextrin	516.67
Fructose	116.67
Sugar	79.07
Citric acid	1.00
Natural cherry flavor	2.00
Cherry syrup	36.00
Red 40	5.00
Blue color	2.00
**Reconstituted placebo drink (200 mL) [b]**	
pH	3.52 ± 0.06
Brix	18.11 ± 1.17
Titratable acidity (%, as malic acid)	0.12 ± 0.03
Energy (Kcal)	~142
Dietary fiber (g)	-
Protein (g)	-
Fat (g)	-
Phenolic acids (mg gallic acid equivalent)	-
Anthocyanins (mg C3G)	

[a] Maltodextrin (Maltrin M180; Muscatine, IA), fructose (ADM; Decatur, IL), granulated sugar ("Great Value"; Bentonville, AZ), citric acid ("Milliard"; Lakewood, NJ) natural cherry flavor ("Bell", Northbrook. IL), cherry syrup ("Torani," San Leandro, CA), Red 40 and Blue 1 ("McCormick", Hunt Valley, MD) [b] Data are presented as mean ± SEM

Supplementary Table S3. Demographic characteristics of study participants.

Characteristics	Cherry (*n* = 19)	Placebo (*n* = 21)
**Gender**		
Female	11	14
Male	8	7
**Race**		
White	11	15
Black/African American	4	3
Asian	3	2
American Indian/Alaska Native	1	1
**Ethnicity**		
Hispanic	3	2
Not Hispanic	16	19
**Income**		
Less than $10,000	2	2
10,000-24,999	4	0
25,000-49,999	5	3
50,000-74,999	1	5
75,000-99,999	0	4
100,000-150,000	3	2
150,000 and greater	1	1
Prefer not to answer	3	3
**Job Category**	**(*n* = 7)**	**(*n* = 11)**
Student	3	1
Employed	3	8
Housewife	1	0
Self-employed	0	1
Retired	0	1
**Marital status**	**(*n* = 7)**	**(*n* = 10)**
Single	2	3
Married	3	5
Divorced	2	1
Widow	0	1

Supplementary Table S4. Nutritional patterns and HEI scores in cherry and placebo groups

Parameter	Cherry (*n* = 19)	Placebo (*n* = 21)	p-value
Energy (Kcal/day)	1911 (1599, 2224)	2025 (1780, 2269)	0.54
Carbohydrate (gr.)	212.1 (177.4, 246.7)	214.5 (184.9, 244.1)	*0.74*
Fat (gr.)	78.9 (57.5, 100.3)	81.2 (67.5, 94.8)	*0.77*
Protein (gr.)	77.2 (62.9, 91.4)	86.2 (71.1, 101.2)	*0.34*
Fiber (gr.)	12.8 (11.0, 14.5)	16.4 (12.5, 20.2)	*0.16*
Cholesterol (mg.)	284.7 (210.4, 359.1)	259.7 (188.7, 330.7)	*0.52*
HEI Score	40.7 (36.7, 44.7)	42.6 (39.1, 46.1)	0.45

Values are mean (95% CI). Data was checked for normality and log-transformed if needed to perform the unpaired *t*-test. Italicized p-values were obtained from log-transformed data. HEI-healthy eating index

Supplementary Table S5. Analysis of Δ SBP and Δ DBP by BMI between cherry and placebo groups

Variable	BMI level	Treatment		Difference (Cherry- Placebo)	
		Cherry	Placebo	Mean (95% CI)	p value
Δ SBP[#]	**High BMI**	-7.57 (-13.03, -2.10) *n* = 7	3.45 (-4.35, 11.25) *n* = 6	-11.02 (-19.16, -2.88)	**0.008**
Δ DBP[*]	**High BMI**	-6.48 (-12.61, -0.35) *n* = 7	0.28 (-6.35, 6.92) *n* = 6	-6.77 (-15.76, 2.21)	0.13

[#] Data are mean (95% CI). Difference between treatments was determined by unpaired t-test. [*] Data are estimated marginal means (95% CI) obtained after adjustment for significant D1 values. Difference between treatments was assessed by Mann-Whitney test.

Supplementary Table S6. Analysis of Δ IFNγ by BMI and gender between cherry and placebo groups

Variable	Level	Treatment		Difference (Cherry- Placebo)	
		Cherry	Placebo	Mean ± SE	p value
**BMI**	**High**	-0.78 (-1.46, -0.10) *n* = 7	3.92 (-0.37, 8.22) *n* = 4	-4.71 (-7.07, -2.34)	**0.006**
**Gender**	**Male**	-0.41 (-1.22, 0.40) *n* = 7	2.28 (0.59, 3.97) *n* = 6	-2.69 (-4,24, -1.14)	**0.01**

Data are mean (95% CI). Difference between treatments was determined by Mann-Whitney test.

Supplementary Table S7. Differential WBC count in cherry and placebo groups.

Variable	Day	Treatment		2-way ANOVA *p* values		
		**Cherry (*n* = 19)**	**Placebo (*n* = 21)**	**Treatment**	**Day**	**Interaction**
WBC x10^3/uL	D1	6.78 (5.69, 7.87) *n* = 14	6.49 (5.50, 7.48 *n* = 16	*0.38*	*0.94*	*0.62*
	D30	5.93 (3.81, 8.03) *n* = 14	6.34 (5.64, 7.03) *n* = 16			
Neutrophils (%)	D1	52.35 (47.39, 57.31) *n* = 14	53.87 (49.82, 57.92) *n* = 16	0.48	0.95	0.77
	D30	51.92 (44.05, 59.35) *n* = 14	54.50 (50.66, 58.33) *n* = 16			
Lymphs (%)	D1	36.71 (32.33, 41.09) *n*= 14	35 (31.56, 38.93) *n* = 16	*0.38*	*0.48*	*0.58*
	D30	37.21 (30.01, 44.41) *n* = 14	33.37 (29.37, 37.37) *n* = 16			
Monocytes (%)	D1	7.85 (6.84, 8.86) *n* = 14	7.62 (6.27, 8.97) n = 16	0.95	0.84	0.75
	D30	7.78 (6.93, 8.63) *n* = 14	7.95 (6.31, 9.59) *n* = 16			
Eos (%)	D1	2.00 (1.40, 2.59) *n* = 14	2.06 (1.46, 2.66) *n* = 16	*0.80*	*0.79*	*0.60*
	D30	2.28 (1.59, 2.98) *n* = 14	2.25 (1.54, 2.96) *n* = 16			
Neutrophils (Absolute) x10^3/uL	D1	3.66 (2.79, 4.53) *n* = 14	3.53 (2.92, 4.13) *n* = 16	0.62	0.81	0.75
	D30	3.78 (2.90, 4.66) *n* = 14	3.51 (2.99, 4.02) *n* = 16			
Lymphs (Absolute) x10^3/uL	D1	2.40 (2.10, 2.69) *n* = 14	2.39 (1.93, 2.85) n = 16	*0.15*	*0.46*	*0.26*
	D30	2.46 (2.16, 2.76) *n* = 14	2.08 (1.82, 2.34) *n* = 16			
Monocytes (Absolute) x10^3/uL	D1	0.52 (0.43, 0.61) *n* = 14	0.48 (0.40, 0.56) *n* = 16	0.52	0.48	0.75
	D30	0.53 (0.43, 0.63) *n* = 14	0.51 (0.44, 0.59) *n* = 16			
Eos (Absolute) x10^3/uL	D1	0.13 (0.08, 0.18) *n* = 14	0.13 (0.11, 0.16) *n* = 16	*0.62*	*0.16*	*0.53*
	D30	0.17 (0.11, 0.23) *n* = 14	0.15 (0.11, 0.19) *n* = 16			

Data are mean (95% CI). Data was analyzed by fitting a 2-way ANOVA analysis, followed by Šídák multiple comparison test. Italicized *p* values were obtained from log-transformed data. WBC: white blood count.

Supplementary Table S8. Correlation matrix between variables showing significant changes in cherry and placebo groups

Variables	Cherry				
	Δ BW	Δ BMI	Δ IL-10	Δ IFNγ	Δ TG
Δ BW	-	r= 0.66<br>p=0.002	r=0.48<br>p=0.05	-	-
Δ SBP	r=0.67<br>p=0.002	r=0.45<br>p=0.05	-	r=0.48<br>p=0.04	-
	**Placebo**				
Variables	Δ BW	Δ BMI	Δ SBP	Δ IFNγ	Δ TG
Δ BMI	r=0.91<br>p=<0.0001	-	-	-	-
Δ DBP	-	-	r=0.64<br>p=0.002	-	r=0.41<br>p=0.05
Δ IL-1RA	r=-0.43<br>p=0.05	r=-0.51<br>p=0.02	r=0.45<br>p=0.04	-	-
Δ IL-10	-	-	-	r=0.81<br>p=0.0001	-

Spearman's correlation coefficient (r) and p values were obtained using GraphPad Prism (v. 9.3.1)

References

1. F. Abderrahim, E. Huanatico, R. Repo-Carrasco-Valencia, S. M. Arribas, M. C. Gonzalez and L. Condezo-Hoyos, Effect of germination on total phenolic compounds, total antioxidant capacity, Maillard reaction products and oxidative stress markers in canihua (Chenopodium pallidicaule), *Journal of Cereal Science*, 2012, **56**, 410-417.
2. N. N. Lage, M. A. A. Layosa, S. Arbizu, B. P. Chew, M. L. Pedrosa, S. Mertens-Talcott, S. Talcott and G. D. Noratto, Dark sweet cherry (Prunus avium) phenolics enriched in anthocyanins exhibit enhanced activity against the most aggressive breast cancer subtypes without toxicity to normal breast cells, *Journal of Functional Foods*, 2020, **64**, 103710.
3. M. M. Giusti and R. E. Wrolstad, Characterization and Measurement of Anthocyanins by UV-Visible Spectroscopy, *Current Protocols in Food Analytical Chemistry*, 2001, **00**, F1.2.1-F1.2.13.
